# 

**Published:** 1881-04

**Authors:** 


MEETINGS OF STATE DENTAL SOCIETIES IN MAY.—New York, 11th, Albany; Georgia, 10th, Savannah; Iowa, 9th, Davenport; Illinois, 10th, Rock Island; Texas, 4th, Austin; Minnesoto, 26th, Winona; South Carolina, 3d, Cheraw; Kansas, 3d, Topeka.

*We take the privilege of deciding what we shall publish, but the author only is responsible for his production.*

*From this date the **Journal** will not be continued to anyone who does not comply with our terms, viz:*

***$3.00 Per Annum,***

*[$2.75 For Vol. II, 1881,]*

***in Advance.***

AN EXTRA
InducemenT
To Subscribers. See the Advertisement of the "Johnson Revolving Book Case in this Journal.

This column will be sold for advertising space six month during the year at *special rates.*

Money is at OUR RISK ONLY when sent by Post Office Money Order, Registered Letter, or Check on Banks in New York City or Baltimore, made payable to Dr. B. M. Wilkerson, 68 N. Charles street, Baltimore, Md.

If you prefer to send check on your private bank, unless located in the cities of [illegible]

